# Analysis Shiga Toxin-Encoding Bacteriophage in a Rare Strain of Shiga Toxin-Producing *Escherichia coli* O157:H7 *stx2a/stx2c*

**DOI:** 10.3389/fmicb.2020.577658

**Published:** 2020-10-21

**Authors:** David R. Greig, Amy F. W. Mikhail, Timothy J. Dallman, Claire Jenkins

**Affiliations:** ^1^National Infection Service, Public Health England, London, United Kingdom; ^2^Division of Infection and Immunity, The Royal (Dick) School of Veterinary Studies, The Roslin Institute, The University of Edinburgh, Easter Bush, United Kingdom

**Keywords:** Shiga toxin-producing *E. coli*, outbreak, genomics, public health, epidemiology, coconut

## Abstract

In December 2015, six cases of Shiga toxin (Stx)-producing *Escherichia coli* (STEC) O157:H7 *stx2a/stx2c* phage type (PT) 24 were identified by the national gastrointestinal disease surveillance system at Public Health England (PHE). Frozen grated coconut imported from India was implicated as the vehicle of infection. Short and long read sequencing data were interrogated for genomic markers to provide evidence that the outbreak strain was from an imported source. The outbreak strain belonged to a sub-lineage (IIa) rare in domestically acquired infection in the United Kingdom, and indicative of an imported strain. Phylogenetic analysis identified the most closely related isolates to the outbreak strain were from cases reporting recent travel not to India, but to Uganda. Phylo-geographical signals based on travel data may be confounded by the failure of local and/or global monitoring systems to capture the full diversity of strains in a given country. This may be due to low prevalence strains circulating in-country under the surveillance radar, or a recent importation event involving the migration of animals and/or people. Comparison of *stx2a*-encoding prophage harbored by the outbreak strain with publicly available *stx2a*-encoding prophage sequences revealed that it was most closely related to *stx2a*-encoding prophage acquired by STEC O157:H7 that caused the first outbreak of STEC-hemolytic uremic syndrome (HUS) in England in 1982–83. Animal and people migration events may facilitate the transfer of *stx2a*-encoding prophage from indigenous STEC O157:H7 to recently imported strains, or vice versa. Monitoring the global transmission of STEC O157:H7 and tracking the exchange of *stx2a*-encoding phage between imported and indigenous strains may provide an early warning of emerging threats to public health.

## Introduction

Outbreaks of foodborne, gastrointestinal disease caused by Shiga toxin (Stx)-producing *Escherichia coli* (STEC) serotype O157:H7 are regarded as a significant threat to public health (Riley et al., 1983; Michino et al., 1999; Cowley et al., 2016; Launders et al., 2016a; Gobin et al., 2018). A subset of vulnerable patients at the extremes of age are at risk of developing hemolytic uremic syndrome (HUS), a condition characterized by renal failure, and cardiac and neurological complications that can be fatal (Tarr et al., 2005; Launders et al., 2016a). STEC O157:H7 is zoonotic and can be transmitted to humans via direct contact with animals and/or their environment, contaminated food or water, or person-to-person spread (Byrne et al., 2015).

There are three main lineages of STEC O157:H7 (I, II, and I/II) and eight sub-lineages (Ia, Ib, Ic, IIa, IIb, IIc, I/IIa, and I/IIb) (Dallman et al., 2015a). With respect to the clade typing scheme proposed by Iyoda et al. (2014), as described previously, lineage I corresponded to clades 1 through 6, lineage II corresponded to clade 7, and lineage I/II corresponded to clade 8 (Eppinger et al., 2011; Dallman et al., 2015a). The majority of human cases and outbreaks of STEC O157:H7 in the United Kingdom are caused by sub-lineages Ic, I/IIa, and IIc (Dallman et al., 2015a). The sub-lineages that are infrequently isolated from human cases in the United Kingdom are more likely to be associated with returning travelers or outbreaks associated with contaminated food imported to the United Kingdom from elsewhere (Gobin et al., 2018).

The defining pathogenicity factor for STEC O157:H7 is the presence of Stx-producing genes (*stx*). There are two types of Stx, Stx1 and 2, and at least 10 subtypes (1a–1d and 2a–2i) (Scheutz et al., 2012). The genes encoding the *stx* subtypes are located on mobile genetic elements (bacteriophages) that may be acquired by STEC and integrated into the genome. STEC O157:H7 harboring the *stx2a*-encoded phage are most commonly associated with causing HUS (Persson et al., 2007; Byrne et al., 2020).

In December 2015, six cases of STEC O157:H7 phage type (PT) 24 were identified by the national gastrointestinal disease surveillance system at Public Health England (PHE). Following the analysis of the trawling questionnaires, a specific brand of imported frozen grated coconut was implicated as the vehicle of infection. In this study, we conducted an epidemiological investigation of the outbreak, and analyzed short and long read sequencing data to look for genomic markers to provide further evidence that the outbreak strain was from a non-domestic (non-UK) source.

## Materials and Methods

### Microbiological and Epidemiological Data Collection

In England, all fecal specimens from hospital and community cases of gastrointestinal disease submitted to local hospital laboratories are tested for *E. coli* O157:H7. All isolates are submitted to the Gastrointestinal Bacterial Reference Unit (GBRU) at PHE for identification and phage typing. Since July 2015, all isolates have been whole genome sequenced for routine surveillance (National Center for Biotechnology Information Short Read Archive BioProject PRJNA315192).

The majority of isolates of STEC O157:H7 included in this study were submitted to GBRU between July 2015 (when WGS was first implemented) and December 2016 (12 months after the outbreak occurred). During this time frame, 1103 isolates were submitted to GBRU; 149 belonged to household outbreak, 283 were linked to known outbreaks, and 671 were identified as sporadic cases. Additional isolates sequenced from the archive collection submitted to GBRU between 2006 and 2016, included eight isolates belonging to the same PT as the outbreak strain, PT24, all of which reported recent (within 7 days of onset of symptoms) travel to Uganda, and all cases reporting recent travel to the Indian Sub-Continent (ISC; *n* = 56) (Supplementary Table S1).

### Epidemiological Data Collection

In January 2009, PHE implemented the National Enhanced Surveillance System for STEC (NESSS) in England. This system has been described in detail previously (Byrne et al., 2015). Briefly, it captures standardized epidemiological on all cases of STEC reported in England through an Enhanced Surveillance Questionnaire (ESQ) including detailed demographic, clinical, and exposure data which is reconciled with microbiological data in NESSS. Following analysis of the ESQ data, each case was re-interview using a standardized trawling questionnaire.

### Short Read Sequencing on the Illumina HiSeq 2500

Genomic DNA was extracted from cultures of STEC O157:H7 using the Qiagen Qiasymphony (Qiagen, Hilden, Germany). The sequencing library was prepared using the Nextera XP kit (Illumina, San Diego, CA, United States) for sequencing on the *Illumina HiSeq 2500* (Illumina, San Diego, CA, United States) instrument run with the fast protocol. High quality trimmed (leading and trailing trimming at < Q30 using Timmomatic v0.27 (Bolger et al., 2014). Illumina FASTQ reads were mapped to the Sakai STEC O157 reference genome (NC 002695.1) using BWA MEM v0.7.13 (Li and Durbin, 2010) and Samtools v (Li et al., 2009). Variant positions were identified by GATK v2.6.5 UnifiedGenotyper (McKenna et al., 2010) that passed the following parameters: > 90% consensus, minimum read depth of 10, Mapping Quality (MQ) ≥ 30. Any variants called at positions that were within the known prophages in Sakai were masked from further analyses. The remaining variants were imported into SnapperDB v0.2.5 (Dallman et al., 2018).

Hierarchical single linkage clustering was performed on the pairwise single nucleotide polymorphisms (SNPs) difference between all strains at various distance thresholds (250, 100, 50, 25, 10, 5, 0). The result of the clustering is an SNP profile, or SNP address, that can be used to describe the population structure based on clonal groups (Dallman et al., 2015b, 2018). Although isolates greater than 5 SNPs apart are unlikely to be part of the same temporally linked outbreak, deeper phylogenetic relationships within the 10 or 25 SNP clusters may provide epidemiologically useful information or associations. Lineage and sub-lineage assignment were performed based on discriminatory SNPs, extracted directly from SnapperDB v0.2.5, that define the population structure, as described previously (Dallman et al., 2015b, 2018).

### Long Read Sequencing Using ONT and Data Processing

Genomic DNA was extracted and purified using the Qiagen Genomic Tip, midi 100/G (Qiagen, Hilden, Germany) with minor alterations including no vigorous mixing steps (performed by inversion) and elution into 100 μl. DNA was quantified using a Qubit and the high sensitivity (HS) dsDNA Assay Kit (Thermofisher Scientific, Waltham, MA, United States) to manufacturer’s instructions. Library preparation was performed using the Native Barcoding kit (SQK-LSK108 and EXP-NBD103) (Oxford Nanopore Technologies, Oxford, United Kingdom). The prepared library was loaded on a FLO-MIN106 R9.4.1 flow cell (Oxford Nanopore Technologies, Oxford, United Kingdom) and sequenced using the MinION for 24 h.

Data produced in a raw FAST5 format were basecalled into FASTQ format and de-multiplexed using Guppy v2.3.5 (Oxford Nanopore Technologies). The reads were then de-multiplexed again using Deepbinner v0.2.0 (Wick et al., 2018) to reduce barcode contamination. Run metrics were generated using Nanoplot v1.8.1 (De Coster et al., 2018). The barcode and y-adapter from each sample’s reads were trimmed, and chimeric reads split using Porechop v0.2.4. Finally, trimmed reads were filtered using Filtlong v0.1.1 with the following parameters; min_length = 1000, keep_percent = 90, and target_bases = 275Mb, to generate approximately 50x coverage of the STEC genome with the longest and highest quality reads.

### *De novo* Assembly, Polishing, Reorientation, and Annotation

Trimmed ONT FASTQ files were assembled using Canu v1.7.1 (Koren et al., 2017). Polishing of the assembly was performed in a three-step process first, using Nanopolish v0.11.1 (Loman et al., 2015) using both the trimmed ONT FASTQs and FAST5s for each respective sample accounting for methylation using the –methylation-aware = dam, dcm and –min-candidate-frequency = 0.1. Secondly, Pilon v1.22 (Walker et al., 2014) using Illumina FASTQ reads as the query dataset with the use of BWA v0.7.17 (Li and Durbin, 2010) and Samtools v1.7 (Li et al., 2009). Finally, Racon v1.2.1 (Vaser et al., 2017) also using BWA v0.7.17 and Samtools v1.7 (Li et al., 2009) was used with the Illumina reads to produce a final assembly for each of the samples. As the chromosome from the assembly was closed, it was re-orientated to start at the *dnaA* gene (NC_000913) from *E. coli* K-12, using the –fixstart parameter in circlator v1.5.5 (Hunt et al., 2015). Prokka v1.13 was used to annotate the draft assembly (Seemann, 2014).

### Prophage Detection, Excision, and Processing

Prophages across both samples were detected and extracted using the Phage Search Tool (PHASTER) (Arndt et al., 2016). Prophage extraction from the genome occurred regardless of prophage size or quality and any detected prophages separated by less than 4 kbp were conjoined into a single phage using Propi v0.9.0 as described in Shaaban et al. (2016). From here the prophages were manually trimmed to remove any non-prophage genes and were again annotated using Prokka v 1.13 with the use of a personalized database (Amino Acid multi-FASTA) containing known STEC prophage genes was used to annotate the final assemblies. Database publicly available from https://github.com/gingerdave269/prophage_DB. The output GenBank (gbk) files were modified to color genes by function.

### Mash and Phylogeny

Mash v2.2 (Ondov et al., 2016) was used to sketch (sketch length 1000, kmer length, 21) *stx*-encoding prophages from the outbreak isolate sequenced using ONT in this study, and the publicly available STEC genomes listed in Table 4. The pairwise Jaccard distance between the prophages was calculated and a neighbor joining tree computed and visualized using FigTree v1.4.4.

### Visualization Tools

All gene diagrams were constructed using Easyfig v2.2.3 (Sullivan et al., 2011). Neighbor joining trees were visualized and annotated using FigTree v1.4.4.

### Data Deposition

Illumina FASTQ files for all samples used in the study can be found under BioProject PRJNA315192. Nanopore FASTQ file is available under SRA accession: SRR10177137. The assembly can be found under the following accession: CP044350. All the above are available from BioProject: PRJNA315192.

## Results and Discussion

### Epidemiological Investigations

In December 2015, the Gastrointestinal Bacteria Reference Unit identified six cases of STEC O157:H7 with a rare PT, PT24. Whole genome sequencing results confirmed that the cases belong to the same 5-SNP single linkage cluster. The outbreak strain belonged to sub-lineage IIa and had the *stx* profile *stx2a* and *stx2c*.

An outbreak control team was convened, and trawling questionnaires were completed for each case and their symptomatic household contacts. Of the six cases, two were male and four were female, and the age range for all six cases was 1–45 years old. Dates of onset of symptoms of gastrointestinal infection fell between 17 and 25 November 2015. The cases were national distributed. Analysis of the trawling questionnaires identified consumption of frozen grated coconut belonging to the same brand was reported by four of the six cases. Of the remaining two cases, one patient recalled eating coconut yogurt, while the other reported the consumption of Indian sweets and attended a Diwali party in the days before onset of symptoms but did not recall specifically eating coconut.

Microbiological analysis of an unopened packet of the coconut product from the restaurant where two of the cases ate, detected unsatisfactory levels of *E. coli* (>10^2^ cfu/g), as defined by Regulation (EC) No. 178/20021^1^, although no STEC O157:H7 was detected in the sample.

### Phylogenetic Analysis of WGS Data Linked to Patients Travel History to Determine a Domestic or Non-domestic Origin for the Outbreak Strain

Following the epidemiological investigation that provided evidence that the contaminated food vehicle may be imported, we analyzed the genome data to look for evidence that the outbreak strain had a non-domestic origin.

Of the 671 sporadic isolates of STEC O157:H7 referred to GBRU between July 2015 and December 2016, the majority belonged to sub-lineages IIc (246/671, 36.7%) and Ic (183/671, 27.2%) (Table 1). In contrast, sub-lineage IIa was less frequently isolated from sporadic cases (98/671, 14.6%), and showed the highest level of sub-lineage diversity, as measured by the number of different 250-SNP single linkage clusters within each sub-lineage (Table 1). High levels of sub-lineage diversity are representative of infrequent sampling from a geographically dispersed reservoir (Gobin et al., 2018).

**TABLE 1:** Diversity and frequency of sampling within each sub-lineage 2015–2016.

**Sub-lineage**	**Total number of isolates**	**Number of sporadic isolates**	**Number of clusters detected at the 250 SNP level**
Ia	31	29	9
Ib	25	25	4
Ic	234	183	4
IIa	325	98	28
IIb	139	44	4
IIc	285	246	1
I/II	37	24	3
NSF	6	4	2
SF	21	18	5
Total	1103	671	

*NSF, non-sorbitol fermenting STEC O157:H7; SF, sorbitol fermenting STEC O157:H7.*

**FIGURE 1:**
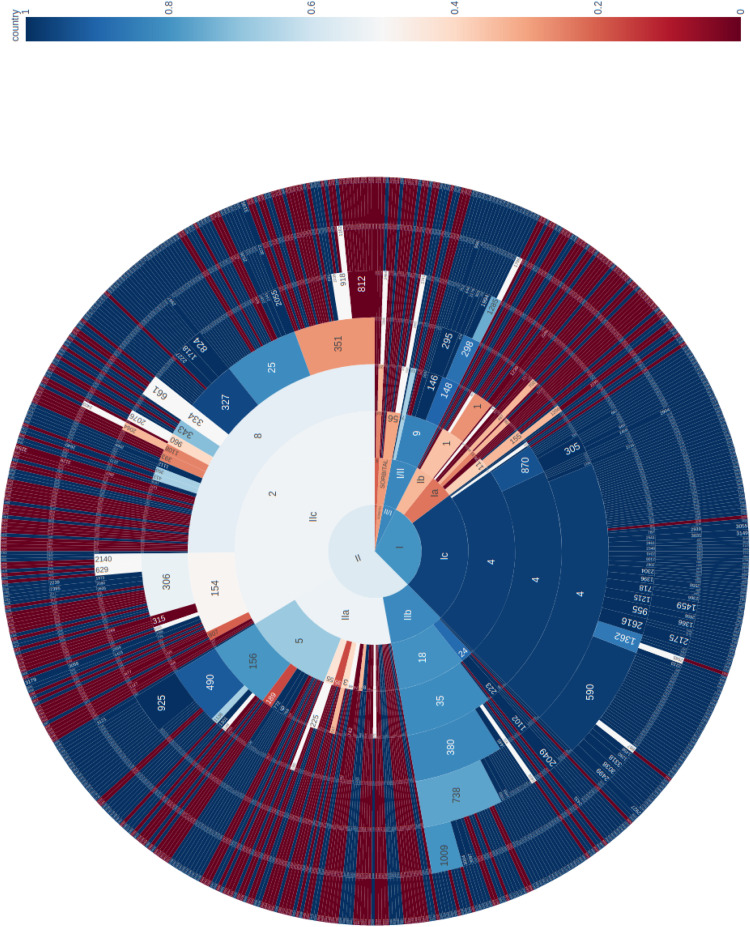
Sunburst diagram showing the distribution of isolates belonging to lineage and sub-lineage, and six descending concentric circles represent single linkage SNP clusters at the 250 SNP, 100 SNP, 50 SNP, 25 SNP, 10 SNP, and 5 SNP levels. The segments were colored based on the proportion of isolates from cases reporting foreign travel with 7 days of onset of symptoms, with dark blue being no cases report recent travel and dark red being all cases report recent travel outside the United Kingdom. For example, within sub-lineage IIa, with the exception of one 50-SNP level cluster (t50: 5.156.490) that was almost exclusively domestically acquired, the majority of the remaining clusters were travel associated.

The phylogeny of the 671 sporadic isolates included in this study were visualized using a sunburst diagram showing the distribution of isolates belonging to lineage and sub-lineage, and six descending single linkage SNP clusters at the 250 SNPs, 100 SNPs, 50 SNPs, 25 SNPs, 10 SNPs, and 5 SNPs level (Figure 1) (Dallman et al., 2018). In Figure 1, the inner circle shows the proportions of the three main lineages and the proportion of those isolates that fall outside the lineage structure. Moving outward from the inner circle, the second concentric circle shows the proportion of isolates in each sub-lineage, and the third, fourth, fifth, sixth, and seventh concentric circles from the inner circle represent the 250 SNPs, 100 SNPs, 50 SNPs, 25 SNPs, 10 SNPs, and 5 SNPs levels, respectively. The numbers represent SNP type or SNP address designation, for example, the SNP address designation for the largest 25 SNP single linkage cluster in sub-lineage IIa is t25: 5.156.490.925%.

The segments were colored based on the proportion of isolates from cases reporting foreign travel with 7 days of onset of symptoms, with dark blue being no cases report recent travel and dark red being all cases report recent travel outside the United Kingdom. The majority of clusters that belong to sub-lineages Ic, IIb, and I/II were domestically acquired. These data were consistent with previous studies that indicated these sub-lineages were likely to be endemic in the United Kingdom (Dallman et al., 2015a; Adams et al., 2016; Byrne et al., 2018). Sub-lineages IIa and IIc displayed a mixture of domestically acquired and travel associated clusters (Figure 1). However, within each sub-lineage, at more discriminatory SNP levels, clear cluster associations to either travel or domestic-acquisition were observed. For example, within sub-lineage IIa, with the exception of one 50-SNP level cluster (t50: 5.156.490.) that was almost exclusively domestically acquired, the majority of the remaining clusters were travel associated. The outbreak strain described in this study fell within the travel-associated clusters providing evidence that the contaminated food vehicle was most likely from an imported source.

### Analysis of WGS Data of Isolates From UK Residents Reporting Recent Travel to the Indian Sub-Continent

**FIGURE 2:**
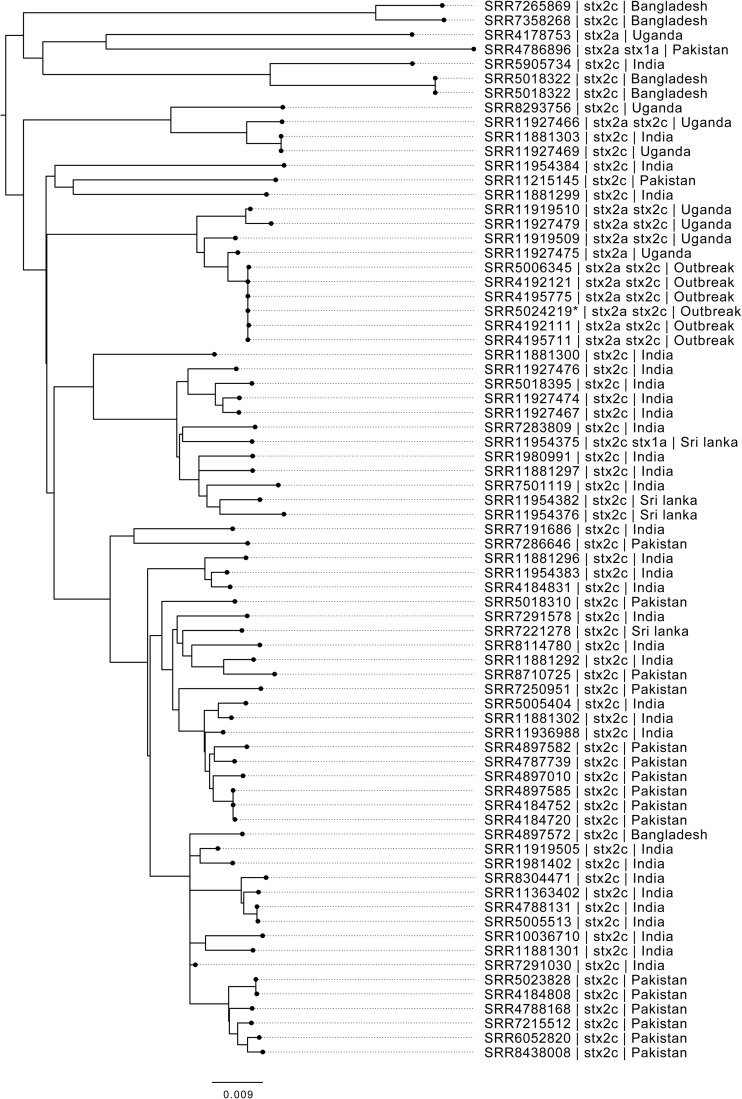
Maximum likelihood phylogenetic tree of strains belonging to sub-lineage IIa, most closely related to the outbreak cluster, showing SRA accession, *stx* type profile, and travel history where available. *denotes Oxford Nanopore Technology sequenced sample 194195.

A closer look at the phylogenetic context of the outbreak strain revealed the most closely related strains were isolates from cases reporting recent travel to Uganda (Figure 2). As the epidemiological investigation indicated that the country of origin of the outbreak strains was India, we included sequences of all the isolates of STEC O157:H7 in the PHE database isolated from travelers recently returned to the United Kingdom from the ISC (Figure 2).

Interrogation of the PHE database of all isolates submitted to GBRU between 2006 and 2016 (*n* = 11,339), identified 56 isolates from UK travelers recently returned from the ISC, the majority reporting recent travel to India (*n* = 32) or Pakistan (*n* = 16) (Table 2). The majority of isolates from the ISC belonged to sub-lineage IIa (53/56), and all had *stx2c* only (*n* = 56). Evidence from studies testing samples from humans, food, and animals in India indicates that the prevalence of STEC O157:H7 is low in this country (Sehgal et al., 2008; Lanjewar et al., 2010; Shrivastava et al., 2017).

**TABLE 2:** Age and sex ratio and characteristics of isolates from UK travelers returning from the ISC and Uganda 2006–2016 (number in parentheses).

**Country**	**Total**	**Male**	**Female**	**Adult**	**Child**	**Lineage (*n*)**	**Stx profile (*n*)**
Bangladesh	4	3	1	2	2	IIa (4)	Stx2c (4)
Bhutan	1	0	1	1	0	Ib (1)	Stx2c (1)
India	32	19	16	24	11	IIa (32)	Stx2c (32)
Maldives	0	–	–	–	–		–
Nepal	0	–	–	–	–		–
Pakistan	16	6	9	2	14	Ia (1) IIa (15)	Stx1a/Stx2a Stx2c (15)
Sri Lanka	3	1	2	2	1	IIa (3)	Stx2c (3)
Total ISC	56	29	29	31	28		
Uganda	8	2	6	6	2	IIa (7) IIb (1)	Stx2a/stx2c (4)

There were eight isolates from UK travelers recently returned from Uganda, all but one belonged to sub-lineage IIa (7/8). Of these, 4/8 had the same *stx* profile (*stx2a*/*stx2c*) as the outbreak strain, and all belonged to a unique clade that had acquired a *stx2a*-encoding bacteriophage. Evidence for the prevalence of STEC O157:H7 in East Africa from human clinical specimens is sparse although STEC O157:H7 has been detected in a number of studies sampling the animal reservoir in this region (Kaddu-Mulindw et al., 2001; Grace et al., 2008; Dulo et al., 2015; Beyi et al., 2017).

Phylo-geographical signals have proved useful in providing evidence for the likely country of origin of STEC and *Salmonella* causing outbreaks of foodborne gastrointestinal disease (Allard et al., 2016; Cowley et al., 2016; Hoffmann et al., 2016; Gobin et al., 2018; Pijnacker et al., 2019). However, there are factors that may confound this signal. Strains of STEC O157:H7 isolated from returning travelers only provide a snapshot of strains endemic to the country visited and may not reflect the full diversity of strains circulating in a given region. Although the outbreak strain may be associated with travel to Uganda, it may also be circulating at low prevalence in India, perhaps due to a recent importation event.

### Analysis of the Outbreak Strain Using Oxford Nanopore Technology

**FIGURE 3:**
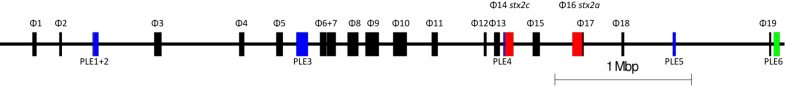
The relative positions of the 19 prophages and six prophage-like elements within the chromosome of 194195. Red prophages indicate *stx*-encoding prophages. Blue indicates prophage like elements. Green indicates the locus of enterocyte effacement (LEE).

We analyzed long read sequencing data of the outbreak strain, primarily to determine the sequence and genomic structure of the *stx2*-encoding bacteriophage. The outbreak isolate assembled into a chromosome of 5,753,376 bp and a single plasmid, pO157 (111,625 bp, IncFIB). The genome of the outbreak isolate comprised 19 prophages (Table 3 and Figure 3).

**TABLE 3:** Size, position, and integration sites of all 19 prophages (P) and six prophage-like elements (PLE) within sample 194195.

**Prophage detected by PHASTER**	**Gene 5’ to prophage**	**Gene 3’ to prophage**	**Size (bp)**	**Position**
P1	*cpxP*^*b*^	*fieF*	31,356	237,188–268,544
P2^*a*^	*zur*	*aphA*	19,508	433,909–453,417
PLE1	*leuX*	Hypothetical^*b*^	9,862	678,235–688,097
PLE2	*intA*^*b*^	*nanM*	33,695	688,557–722,252
P3^*a*^	tRNA-Thr (cgt)	*prgR*	54,474	1,128,970–1,183,444
P4	*ybhC*^*b*^	*ybhB*	38,693	1,751,265–1,789,958
P5	*yccA*^*b*^	tRNA-Ser (tga)	49,561	2,021,966–2,071,527
PLE3	*ycdU*	tRNA-Ser (gga)	85,778	2,170,090–2,255,868
P6^*a*^	*potC*	*potB*	48,949	2,342,248–2,391,197
P7	*potA*	*caeB*^*b*^	65,659	2,393,794–2,459,453
P8^*a*^	*ompW*	*ispE*	79,722	2,544,993–2,624,715
P9^*a*^	*ompW*	*trpA*	98,899	2,675,966–2,774,865
P10^*a*^	*ttcA*	*rspR*	101,788	2,877,961–2,979,749
P11	*ompN*	*rspA*^*b*^	45,568	3,160,400–3,205,968
P12	*yecA*^*b*^	tRNA-Leu (taa)	20,043	3,542,644–3,562,687
P13	*yodB*	tRNA-Ser (cga)^*b*^	43,687	3,617,610–3,661,297
PLE4	*cobU*	Hypothetical	13,348	3,684,489–3,697,837
P14 (*stx2c*)	*yeeW*	*sbcB*^*b*^	60,586	3,698,735–3,759,321
P15^*a*^	*yehV*	*mlrA*^*b*^	52,982	3,900,672–3,953,654
P16 (*stx2a*)	*yfcV*	*argW*^*b*^	74,662	4,190,350–4,265,012
P17	*argW*^*b*^	*lacY*	8,233	4,265,444–4,273,677
P18	*ssrA*	*alpA*^*b*^	20,904	4,549,827–4,570,731
PLE5	tRNA-Phe (gaa)	*pitB*	23,331	4,924,934–4,948,265
P19	*yicC*^*b*^	*yicC*	15,440	5,631,805–5,647,245
PLE6 (LEE)	*selC*	*yicL*	43,021	5,665,860–5,708,881

*^*A*^Refers to prophages that appear to be compound prophages (i.e., two or more prophages that are sequential or one phage integrated inside another, with intact integrase genes). ^*B*^Refers to the end in which the Integrase gene (*intA*) is located.*

**TABLE 4:** Summary of the PHE archived and publicly available strains used within this study, their Strain ID, lineage, phage type, *stx* profile, assembly accession numbers, and NCBI BioProject.

**Strain ID**	**lineage**	**Phage type**	***Stx* profile**	**Number of prophages**	**Reference**	**BioProject #**	**Assembly accession #**
**PHE archive**							
194195	IIa	PT24	*Stx2a/2c*	19	This study	PRJNA315192	CP044350
E30228	Ia	PT4	*Stx1a/2a*	15	Scotland et al., 1987	PRJNA315192	VXJO00000000
E34500	I/IIa	PT2	*Stx2a/2c*	14	Taylor et al., 1986	PRJNA315192	VXJN00000000
E45000	I/IIb	PT49	*Stx2a*	17	Yara et al., 2020	PRJNA315192	VXJM00000000
E116508	Ic	PT21/28	*Stx2a/2c*	17	Yara et al., 2020	PRJNA315192	VXJP00000000
315176	IIb	PT8	*Stx2a*	16	Byrne et al., 2018	PRJNA315192	VXJQ00000000
267849	IIa	PT34	*Stx2a/2c*	16	Gobin et al., 2018	PRJNA315192	VXJR00000000
**Publicly available**							
9000	Ic	PT21/28	*Stx2a/2c*	17	Shaaban et al., 2016	PRJNA336330	CP018252
397404	Ic	PT21/28	*Stx2a/2c*	15	Yara et al., 2020	PRJNA315192	CP043019
155	Ic	PT32	*Stx2a*	18	Shaaban et al., 2016	PRJNA336330	CP018237
350	IIc	PT8	*Stx1a/2c*	16	Launders et al., 2016b	PRJNA336330	CP018243
272	I/IIa	PT2	*Stx2a*	16	Jenkins et al., 2015	PRJNA336330	CP018239
644	IIc	PT8	*Stx1a/2c*	18	Cowley et al., 2016	PRJNA321984	CP015831
180	IIc	PT54	*Stx1a/1a/2c*	15	Cowley et al., 2016	PRJNA321984	CP015832
Sakai	Ia	n/a	*Stx1a/1a/2a*	18	Michino et al., 1999	PRJNA57781	NC_002695
EDL933	Ia	n/a	*Stx1a/2a*	14	Riley et al., 1983	PRJNA253471	CP008957
EC4115	I/IIa	n/a	*Stx2a/2c*	17	Uhlich et al., 2008	PRJNA224116	NC_011353
TW14359	I/IIa	n/a	*Stx2a/2c*	17	Uhlich et al., 2008	PRJNA224116	NC_013008

**FIGURE 4:**
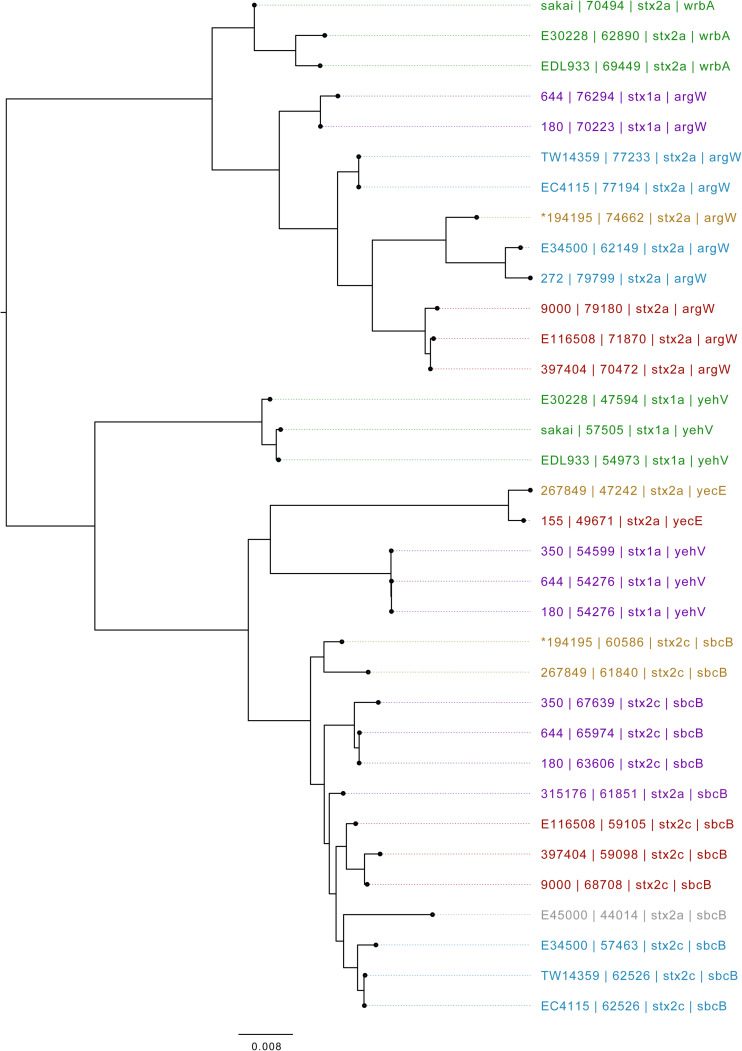
Mid-rooted neighbor joining tree of Shiga toxin-encoding prophages based on Jaccard distance produced from Mash. Strains are annotated as Strain ID, length, and *stx* type profile. Strains are colored by lineage—green: Ia, red: Ic, blue: I/IIa, gray: I/IIb, orange: IIa, purple: IIb. *denotes Oxford Nanopore Technology sequenced sample 194195.

**FIGURE 5:**
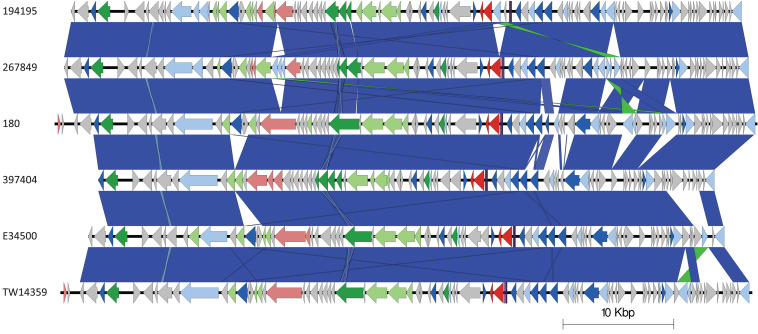
Easyfig plots comparing the *stx2c*-encoding prophages from 194195, 267849, 180, 397404, E34500, and TW14359 in descending order. Arrows indicate gene directions. *stx* genes are shown in red; recombination/replication genes are shown in light blue; regulation-associated genes are shown in dark blue; effector genes are shown in pink; structure- and lysis-associated genes are shown in light and dark green, respectively; tRNAs are shown as purple lines; finally, hypothetical genes are shown in gray.

Based on Mash distance, the *stx2c*-encoding prophage located at the *stx*-encoding bacteriophage insertion (SBI) site *sbcB*, clustered on the same branch as *stx2c* prophage from strains from different time frames, geographical regions, and sub-lineages, most closely related to other *stx2c*-encoding prophage from sub-lineage IIa (Figure 4). The *stx2c*-encoding prophage from the outbreak strains aligned across the length of other *stx2c*-encoding prophage with few structural variations (Figure 5). The conserved nature of the *stx2c*-encoding bacteriophage was consistent with the hypothesis that the *stx2c*-encoding bacteriophage was acquired prior to the global dissemination and regional expansions of STEC O157:H7 (Dallman et al., 2015a).

The *stx2a*-encoding prophage acquired by the outbreak strain was located at SBI site *argW*. Compared to the *stx2c*-encoding prophage, the *stx2a*-encoding prophage exhibits greater diversity, based on Mash distance and whole prophage alignment (Figure 4). In certain countries, the regional expansion of specific sub-lineages, or clades within sub-lineages, has involved acquisition of a stx2a-encoding prophage (Dallman et al., 2015a; Yara et al., 2020).

**FIGURE 6:**
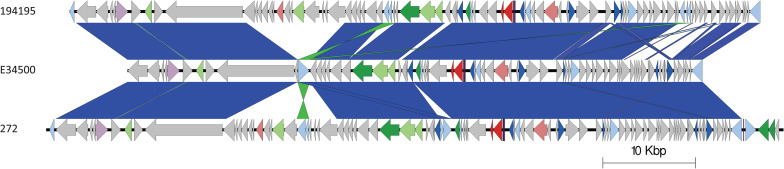
Easyfig plots comparing the *stx2a*-encoding prophages from 194195 with E34500 and 272, in descending order. Arrows indicate gene directions. *stx* genes are shown in red; recombination/replication genes are shown in light blue; regulation-associated genes are shown in dark blue; effector genes are shown in pink; structure- and lysis-associated genes are shown in light and dark green, respectively; tRNAs are shown as purple lines; finally, hypothetical genes are shown in gray.

The *stx2a*-encoding prophage from the outbreak strain was most closely related to *stx2a*-encoding prophage acquired by a strain belonging to lineage I/II that caused the first outbreak of STEC-HUS in the West Midlands in England in the early 1980s (Figures 4, 6) (Taylor et al., 1986; Yara et al., 2020). Previous studies have shown that this lineage I/II strain of STEC O157:H7 harboring *stx2c*-encoding prophage had been indigenous in UK domestic cattle population for decades, and that it emerged as a threat to public health in 1983, following the acquisition of a *stx2a*-encoding prophage at some point during the previous decade (Dallman et al., 2015a; Yara et al., 2020).

## Conclusion

The use of WGS for surveillance of gastrointestinal pathogens enabled us to identify a small, geographically dispersed outbreak of foodborne disease. The epidemiological analysis provided evidence that the outbreak strains originated from India, while the phylogenetic analysis of the sequencing data indicated the strain was most closely related to isolates from Uganda, and the *stx2a*-encoding phage was most closely related to *stx2a-*encoding bacteriophage harbored by the strains of STEC O157:H7 that emerged in the United Kingdom, as the most common cause of STEC-HUS in early 1980s.

These analyses described in this study are open to interpretation in a number of different ways. Microbiological investigation of the grated coconut samples did not detect STEC O157:H7, and the contaminated food vehicle may have been an imported product from elsewhere. Alternatively, the epidemiological evidence indicating India as the country of origin of the outbreak strain may have been correct, but the phylo-geographical signal was obscured by the low prevalence of the outbreak strain in that region. Strains of STEC O157:H7 that have a low prevalence in a specific region may not be captured by either local or global monitoring systems, and it is likely the full diversity of strains in a given region may circulate under the surveillance radar. Moreover, non-indigenous strains of STEC O157:H7 may be introduced into a new region by the migration of animals (including migratory birds) and people, thus further confounding the phylo-geographical signal. These animal and people migration events may facilitate the transfer of mobile genetic elements, such as the *stx2a*-encoding prophage, from indigenous strains of STEC O157:H7 to recently imported strains, or vice versa. Monitoring the transmission of strains of STEC O157:H7 on a global scale, and tracking the exchange of *stx2a*-encoding phage between imported and indigenous strains, may provide an early warning of emerging threats to public health.

## Data Availability Statement

Illumina FASTQ files for all samples used in the study can be found under BioProject PRJNA315192. Nanopore FASTQ file is available under SRA accession: SRR10177137. The assembly can be found under the following accession: CP044350. All the above are available from BioProject: PRJNA315192.

## Ethics Statement

Ethical review and approval was not required for the study on human participants in accordance with the local legislation and institutional requirements. Written informed consent from the participants’ legal guardian/next of kin was not required to participate in this study in accordance with the national legislation and the institutional requirements. The authors declare that there is no requirement for ethical approval for this submission. This work was undertaken to inform the delivery of patient care and to prevent the spread of infection, defined as USUAL PRACTICE in public health and health protection.

## Author Contributions

AM performed epidemiological investigations. DG performed DNA extraction, library preparation, Nanopore sequencing, data processing, genome assembly, correction, and annotation, created Easyfig diagrams, and performed the prophage comparison using Mash with associated scripts designed by TD. TD created sunburst plots. CJ and DG wrote the original manuscript. CJ, DG, and TD reviewed the manuscript. CJ and TD supervised DG. All authors contributed to the article and approved the submitted version.

## Conflict of Interest

The authors declare that the research was conducted in the absence of any commercial or financial relationships that could be construed as a potential conflict of interest.

## Footnotes

1 https://www.gov.uk/government/publications/ready-to-eat-foods-microbiological-safety-assessment-guidelines
